# Liver transplantation for mitochondrial DNA depletion syndrome caused by *MPV*17 deficiency: a case report and literature review

**DOI:** 10.3389/fsurg.2024.1348806

**Published:** 2024-07-11

**Authors:** Liu-Yuan Wei, Xiu-Qi Chen, Li Huang, Qing-Wen Shan, Qing Tang

**Affiliations:** ^1^ Department of Pediatrics, The First Affiliated Hospital of Guangxi Medical University, Nanning, China; ^2^ Department of Pediatrics, The Fourth Affiliated Hospital of Guangxi Medical University, Liuzhou Worker's Hospital, Liuzhou, China

**Keywords:** mitochondrial DNA depletion syndrome, mitochondrial disease, *MPV17* gene, liver transplant, chidren

## Abstract

**Objective:**

To study the effectiveness of liver transplantation (LT) in treating mitochondrial DNA depletion syndrome (MDS) caused by the *MPV*17 gene variant.

**Case presentation:**

A boy aged 2.8 years presented with edema of the lower limbs and abdomen, which persisted for over 10 days and was of unknown origin; this was accompanied by abnormal liver function, intractable hypoglycemia, and hyperlactatemia. During the second week of onset, he developed acute-on-chronic liver failure and was diagnosed with MDS due to homozygous variant c.293C>T in the *MPV*17 gene. Subsequently, he underwent LT from a cadaveric donor. At follow-up after 15 months, his liver function was found to be normal, without any symptoms. Additionally, a literature review was performed that included MDS patients with the *MPV*17 variant who underwent LT. The results demonstrated that the survival rates for MDS patients who underwent LT were 69.5%, 38.6%, 38.6%, and 38.6% at 1-year, 5-year, 10-year, and 20-year intervals, respectively. Sub-group analyses revealed the survival rate of MDS patients with isolated liver disease (83.33%, 5/6) was higher than that of hepatocerebral MDS patients (44.44%, 8/18). Fifteen variants were identified in the *MPV*17 gene, and patients with the c.293C>T (p.P98l) variant exhibited the highest survival rate.

**Conclusion:**

Hepatocerebral MDS patients without neurological symptoms may benefit from LT.

## 1 Introduction

The mitochondrial DNA depletion syndrome (MDS) comprises a group of rare autosomal recessive genetic diseases that cause energy disorders in organs due to the reduction of mitochondrial DNA caused by variants in nuclear genes (1). The manifestation of MDS encompasses a wide range of tissues and organs, with early-onset MDS primarily characterized by predominant involvement in the brain, muscle disorders, and liver. Based on the clinical manifestation, MDS can be classified into myopathic, encephalomyopathic, hepatocerebral, and neurogastrointestinal disorders. Hepatocerebral MDS is associated with four genes: *DGUOK*, *POLG*, *MPV17*, and *C10orf2* (2, 3). The *MPV*17 gene is located on chromosome 2p23.3 and consists of 8 exons that encode a protein that comprises 176 amino acids. This protein, known as the inner mitochondrial membrane protein, plays a crucial role in maintaining mitochondrial DNA and facilitating oxidative phosphorylation in mammals. The MDS causes severe organs disorders, with a poor prognosis in the majority of affected individuals. The therapeutic interventions available for MDS are currently limited, primarily focusing on providing symptomatic management, such as nutritional modulation and supplementation of cofactors, which may be used to slow down disease progression (1, 4–6). LT may be considered for certain patients, although its effectiveness remains a subject of controversy. In this case report, we present a case of LT for the treatment of hepatocerebral MDS with isolated liver disease associated with the *MPV*17 variant. After LT, the patient's liver function was found to remain normal and there were no neurological symptoms.

## 2 Case presentation and literature review

### 2.1 Case presentation

**Figure 1:**
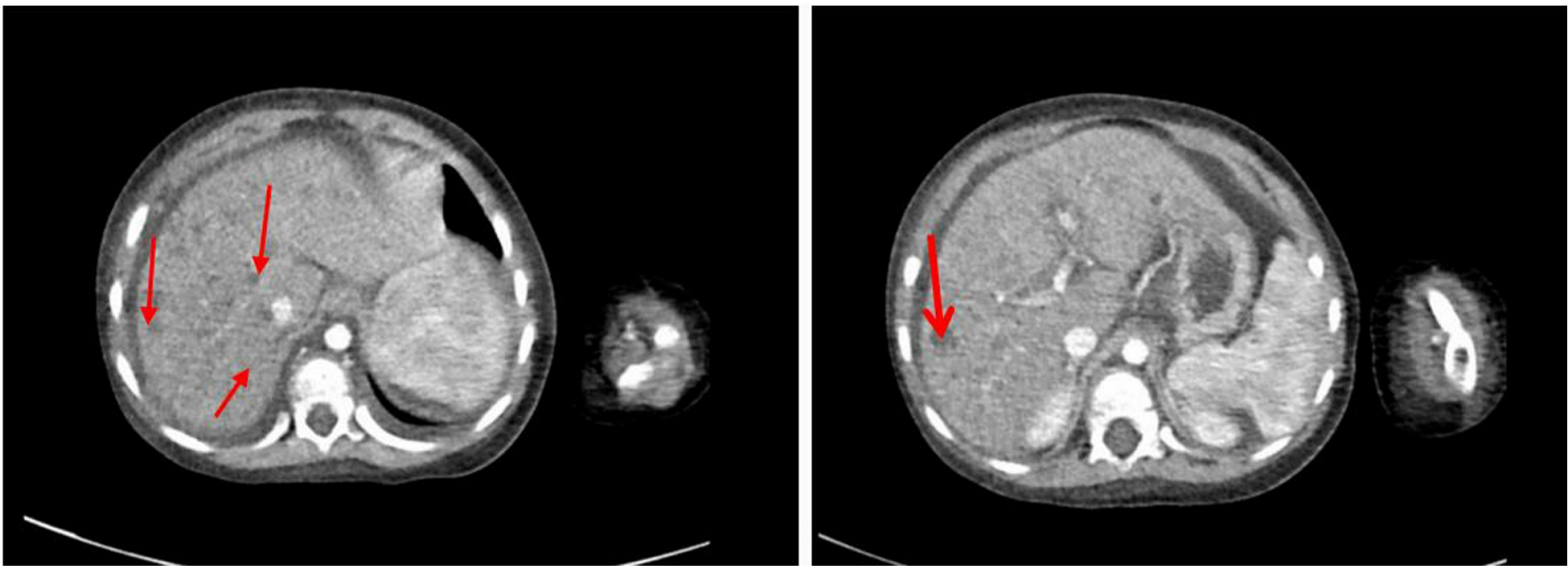
Computerized tomography (CT) of the liver. Multiple low-density foci were found in hepatic parenchyma.

A male patient aged 2.8 years was admitted to our hospital presenting with edema of the lower limbs and abdomen, which had persisted for over 10 days and was of unknown origin. There was no history of persistent vomiting, feeding difficulty, and muscle weakness. However, the patient had a history of failure to thrive. There was no similar history among his family members, and the parents were not consanguineous. The child had a personal history of being G1P1, born at full term through natural birth, with a birth weight of 3,200 g. His psychomotor development progressed typically, with head control achieved at three months, sitting independently at six months, and walking while vocalizing “mom” by one year of age. Language and motor milestones were consistent with developmental norms for children of the same age. Upon physical examination, the patient's vital signs were found to be normal. The patient's body height was 81 cm (<P3), weight was 11.2 kg (P3), and head circumference was 46 cm (P3). The entire body exhibited evident edema, particularly in the lower extremities and eyelids. The patient showed no signs of pallor or icterus. An abdominal examination revealed distension, while palpation of the liver and spleen did not indicate enlargement. Moreover, the examination of the chest, cardiovascular system, and central nervous system revealed unremarkable findings. An abdominal ultrasound revealed a hypoechoic mass of hepatic parenchyma. The laboratory examination revealed that the alanine aminotransferase (ALT), aspartate aminotransferase (AST), and gamma-glutamyl transferase (γ-GGT) were 73 U/L (reference ranges 0 U/L–40 U/L), 209 U/L (0 U/L–40 U/L), and 120 U/L (0 U/L–40 U/L), respectively. The total bile acids (TBA) were 270 µmol/L (reference ranges 0 µmol/L–10 µmol/L), total bilirubin (TBIL) was 46 µmol/L (0 µmol/L–26 µmol/L), direct bilirubin (DBIL) was 33 µmol/L (3.1 µmol/L–14.3 µmol/L), and albumin (ALB) was 21 g/L (40 g/L–55 g/L). The coagulation function revealed that prothrombin time (PT) was 28 s (reference ranges 9 s–15 s), APTT was 47.1 s (reference ranges 23 s–40 s), fibrinogen (Fib) was 0.83 g/L (reference ranges 2 g/L–5 g/L), and INR was 2.3. The blood glucose concentration was 0.6 mmol/L, lactate concentration was 3.81 mmol/L (0 mmol/L–2.5 mmol/L), and blood ammonia concentration was 93 µmol/L (0 mmol/L–72 µmol/L). The concentration of alpha-fetoprotein was 2,677.80 ng/ml. The tumor markers were detected to rule out the presence of primary tumors or metastases, and the results revealed that the glycosyl antigen CA125 was 333.40 U/ml and CA199 was 86.08 U/ml. In addition, the tests for hepatotropic virus, syphilis, human immunodeficiency virus, Epstein-Barr virus, cytomegalovirus, and autoimmune liver disease antibodies were negative. The computed tomography (CT) scan revealed the presence of cirrhosis, multiple hepatic nodules, splenomegaly, and ascites (Figure 1). The acoustic radiation force impulse (ARFI) revealed a significant elevation in liver stiffness, which is indicative of early-stage cirrhosis (30.3 kP, >F4 stage). Further, the results of cardiac ultrasonography and magnetic resonance imaging (MRI) of the brain were normal. However, the liver function and coagulation function of the patient deteriorated progressively after admission.

**Figure 2:**
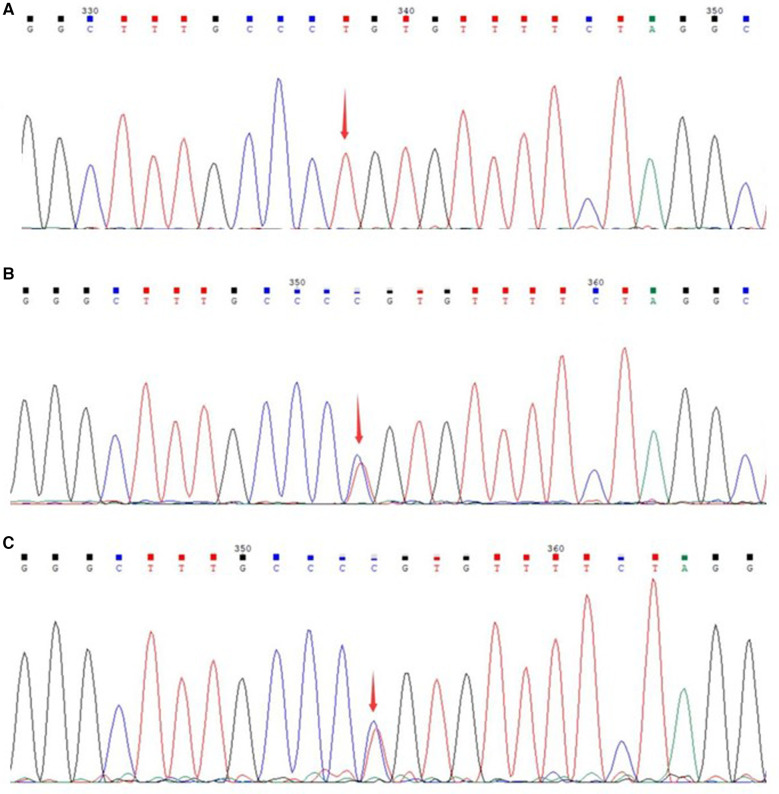
Sanger sequencing of the genes of the patient and parents. (**A**) Gene variant of the patient; (**B**) gene sequencing of the father; (**C**) gene sequencing of the mother.

The patient was initially diagnosed with inherited metabolic liver disease due to refractory hypoglycemia, high lactate levels, and abnormal liver function. After obtaining the guardian's consent, peripheral blood samples were collected from the patient and his parents for whole exome gene analysis. Subsequently Sanger validation was performed. The results revealed the presence of a homozygous missense variant in the *MPV*17 gene c.293C>T (p.P98l) (Figure 2).

**Figure 3:**
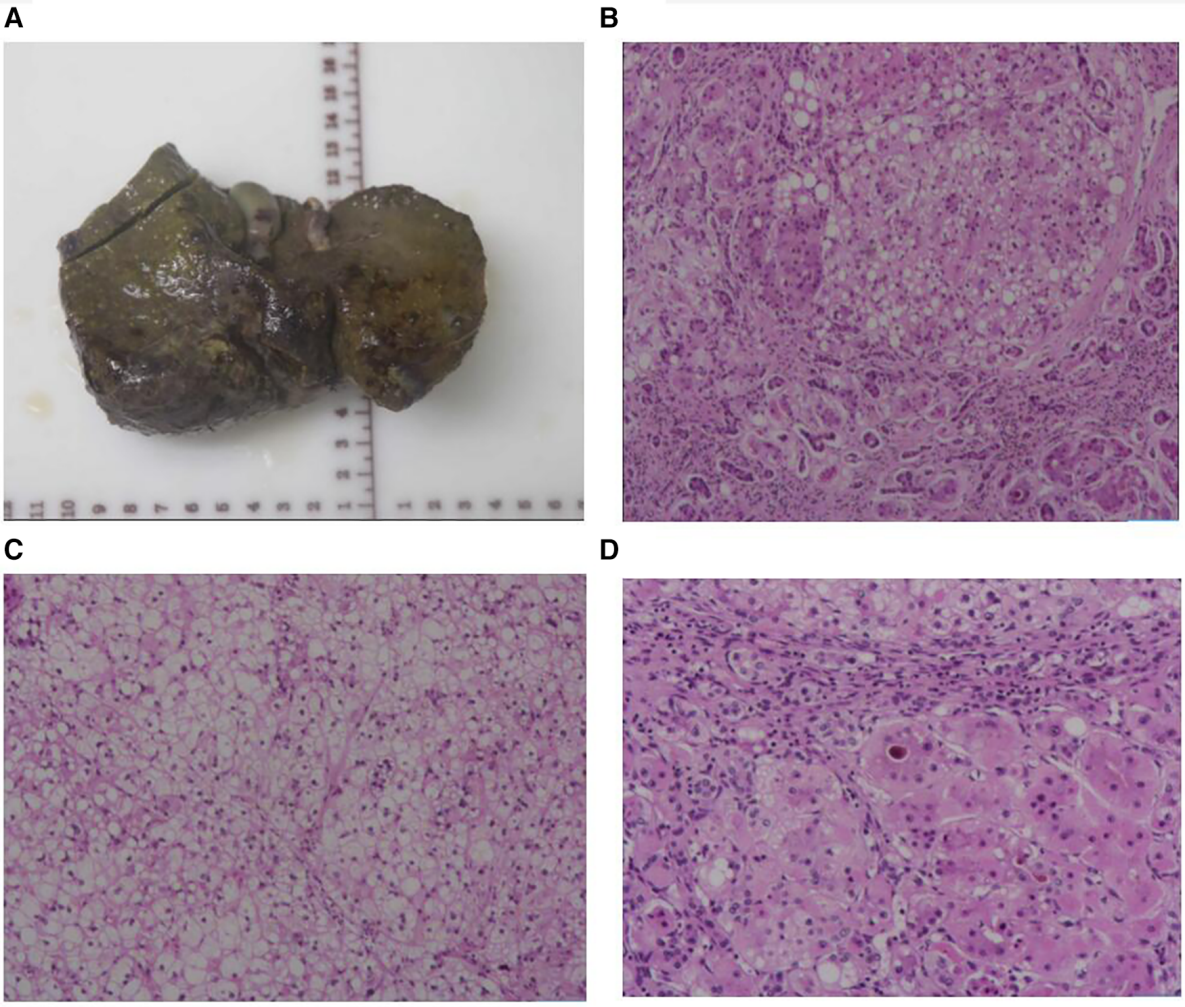
Liver morphology. (**A**) The whole liver tissue is a piece, the size is 12.5 × 9.2 × 5.3 cm, the section surface is gray-brown solid, and the quality is medium, showing nodular cirrhosis. (**B**) Microscopic examination of the structure of the liver lobules disappeared, fibrous tissue proliferation divided the liver lobules to form pseudolobules, small bile duct hyperplasia, and some hepatocyte steatosis. (**C**) Microscopic examination of the lobular structure of the liver disappears, hepatocyte edema, and steatosis. (**D**) Microscopic examination of the liver lobular structure is lost, fibrous tissue is accompanied by more lymphocyte infiltration, and gallbladder stasis can be seen. Liver pathological grading and staging showed that the inflammatory response is G2, fibrosis is S4.

After four weeks of admission (At aged 2.9 years), the patient developed acute-on-chronic liver failure. ALT was 3,507 U/L, AST was 1,733 U/L, GGT was 789 U/L, TBA was 366 µmol/L, TBIL was 507 µmol/L, DBIL was 358 µmol/L, and ALB was 27 g/L. The PT was 42 s, APTT was 59 s, Fib was 1.1 g/L, and the INR value was 3.4. This patient had no hepatic encephalopathy or psychobehavioral abnormalities at the time of liver failure. The patient underwent LT following the standard protocol, using a graft from a deceased donor. The donor, a brain-dead citizen, was registered with the China Human Organ Donation Management Center. Postoperative liver pathology revealed chronic hepatitis and nodular cirrhosis (Figure 3). The blood glucose and lactate concentration returned to normal within the first week following LT, coagulation function normalized 1.5 months after LT, and liver function was restored to baseline within 2 months after LT due to bacterial peritonitis. Tacrolimus and mycophenolate mofetil are currently being used for immunosuppression after LT. After the 12-month follow-up, the child was hospitalized twice due to respiratory infections. However, his blood glucose, lactate concentration, liver function, and coagulation remained within normal ranges. Currently, the patient is aged 3.8 years, with no neurological symptoms. The patient's body height is 102 cm (P25–P50) and weight is 16.5 kg (P50). In addition, his psychomotor development is consistent with the developmental norms for children of the same age.

### 2.2 Literature review

Twenty-three cases of MDS patients with hepatoencephalopathy caused by the *MPV*17 gene variant who underwent LT have been studied in the literature (7–16); 91.3% (21/23) of these patients experienced the onset of the disease within the first year of life. Moreover, isolated liver disease accounted for 21.7% (5/23) of the cases.

Among the 23 patients with LT, 12 survived while 11 died, thereby yielding a survival rate of 52.17% (12/23). Among the five hepatocerebral MDS patients with isolated liver disease, one patient died due to multi-organ failure a month after surgery. The other four patients achieved a survival rate of 80% (4/5), with four patients exhibiting no neurological symptoms during the three-year follow-up after LT. However, one patient developed neurological symptoms after five years and remarkably remained alive for up to 22 years after LT.

From among the 18 hepatocerebral MDS patients for whom the liver and nervous system were involved, 10 patients died (55.56%). The causes of death included sepsis in three cases, multiple organ failure in two cases, pneumonia in two cases, heart failure in two cases, and respiratory failure in one case due to respiratory muscle weakness. The liver failure symptoms disappeared after LT in 88.89% (16/18) of the cases, while neurological symptoms continued to progress.

**Figure 4:**
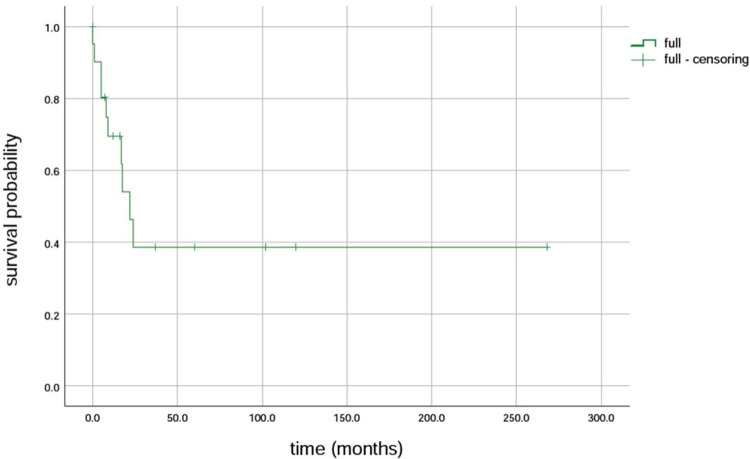
Survival rate of liver transplantation for MPV17 variant-associated mitochondrial DNA depletion syndrome.

Further, 15 variants in the *MPV*17 gene were identified in 23 patients. The first variant was c.149G>A (p.R50Q), found in 43.5% (10/23) of the patients, followed by c.451dupc: p.L151P fs*39, which was found in 26% (6/23) of the patients. Additionally, c.148C>T (p.R50W) was found in 17.4% (4/23) of the patients, and c.293C>T (p.P98l), the fourth variant, was found in 13.0% (3/23) of the patients (7–16). The survival rates of LT with the MPV17-related MDS were firstly c.293C>T (p.P98l) (100% 3/3), followed by c.149G>A (80%, 8/10), and then c.148C>T (50%, 4/8) (Table 1). The results revealed that the median survival time was 22 months, with survival rates of 69.5%, 38.6%, and 38.6% at intervals of 1 year, 5 years, and 10 years, respectively (Figure 4).

**Table 1:** Liver transplantation with the *MPV*17-related MDS.

Gene variant	ACMG	Number of LT	survivors	Age at onset	Isolated liver disease
c.149G>A (p.R50Q)	Pathogenic	10	8	Neonatal – 4 years	2
3 c.293C>T (p.P98l)	Pathogenic	3	3	Neonatal - 2years	1
c.451dupC:p. L151P fs*39	Pathogenic	6	1	1–8 Months	1
c.148C>T (p.R50W)	Pathogenic	4	3	7 Months-4years	2

ACMG, American College of Medical Genetics and Genomics variant classification; MDS, mitochondrial DNA depletion syndrome.

## 3 Discussion

Hepatocerebral MDS caused by the *MPV*17 gene variant exhibits an early onset in infancy; however, it can also manifest at other ages, including in adulthood (17–20). The manifestations of hepatocerebral MDS caused by the *MPV*17 gene variant are primarily found in the hepatic and nervous systems (21). The liver manifestations include cholestasis, hepatomegaly, steatosis, and cirrhosis; the neurological manifestations include muscle weakness and dystonia, sensory deficits, peripheral neuropathy, motor abnormalities, microcephaly, seizures, etc. Additionally, other presentations include metabolic disorders (hyperlactatemia, hypoglycemia, and hyperammonemia) and gastrointestinal symptoms (weight loss/poor weight gain, difficulties in feeding, vomiting, and dysphagia). The liver and nervous system were both involved in 75% of the patients, while isolated liver disease was observed in 25% of the patients.

No effective therapy is currently available for MDS. The existing treatment mainly focuses on providing symptomatic management, which involves the administration of vitamins, respiratory substrates, coenzymes, etc. (1, 22). Increasing the frequency of feeding or considering continuous feeding, along with the administration of raw cornstarch, can be potential strategies to enhance hypoglycemia management (23). However, patients still experience liver degeneration and eventually develop neurodegeneration (24). Gene therapy and nucleoside supplements for MDS are currently in the preclinical phase. In addition, AAV-mediated gene therapy revealed rescue in mtDNA and partial rescue in multiple oxidative phosphorylation function in MDS models carrying mutations of *MPV17* (24–26).

Further, the prognosis for individuals with *MPV*17-associated hepatocerebral MDS is generally poor, as evidenced by the occurrence of early liver failure in 80% of the cases (2). In the absence of LT, most affected individuals succumb to progressive liver failure during infancy or early childhood (27, 28). LT is the only effective intervention for liver failure, yet its efficacy remains contentious due to the involvement of multiple organs. Studies have revealed that although LT can yield a 10-year survival rate in children with MDS, it does not prevent the development of neurological symptoms after LT (21). The results showed a 1-year survival rate of 69.5%, 5-year survival rate of 38.6%, and 10-year survival rate of 38.6% (29).

LT can ameliorate hepatic symptoms and complications, such as coagulation dysfunction, hypoproteinemia, hyperlactatemia, hypoglycemia; however, it does not improve neurological symptoms (7, 16). Study showed that MDS with isolated liver disease before LT; however, they presented neurological symptoms such as epilepsy, mild intellectual disability, dysarthria, and motor dysfunction five years after LT (7). The neurological symptoms continued to progress after LT, with a duration ranging from 1 to 11 years (7–9, 14, 16).

The prognosis may vary for individuals with different *MPV*17 gene variants in LT. The literature data revealed that the c.149G>A (p.R50Q) variant might be associated with a more favorable prognosis, followed by the variant c.293C>T (p.P98l) (23, 30). The mortality rate after LT was 45.8%. It is crucial to emphasize the necessity of preventing infections such as sepsis, pulmonary hypertension, heart failure, respiratory failure, and multiorgan failure following LT. The utilization of LT as a beneficial therapeutic approach can be considered in patients harboring mutations in *DGUOK*, *MPV17*, and *POLG* due to their clinical manifestation of severe hepatic impairment. In addition, isolated liver involvement of *DGUOK*-related MDS was reported in 21.9% of the cases (31). LT was performed in 26 patients with *DGUOK*-related MDS. The median survival was 1.92 years in the overall cohort of transplanted patients, and the mortality rate was 50% after LT. Further, 23% of the patients developed additional neurological symptoms after LT (31). All those outcomes in *DGUOK*-related MD were similar to the *MPV17*-related MD. This intervention holds promise for success if patients present exclusively with significant liver damage without concurrent multisystem dysfunction.

In conclusion, hepatocerebral MDS patients without neurological symptoms may derive the greatest benefit from LT.

## Data Availability

The original contributions presented in the study are included in the article/Supplementary Material, further inquiries can be directed to the corresponding author.
